# Genome-wide analyses of small non-coding RNAs in streptococci

**DOI:** 10.3389/fgene.2015.00189

**Published:** 2015-05-20

**Authors:** Nadja Patenge, Roberto Pappesch, Afsaneh Khani, Bernd Kreikemeyer

**Affiliations:** Institute of Medical Microbiology, Virology, Hygiene and Bacteriology, Rostock University Medical CenterRostock, Germany

**Keywords:** sRNA, *Streptococcus*, array, RNAseq, NGS, virulence, gene regulation, transcriptome

## Abstract

Streptococci represent a diverse group of Gram-positive bacteria, which colonize a wide range of hosts among animals and humans. Streptococcal species occur as commensal as well as pathogenic organisms. Many of the pathogenic species can cause severe, invasive infections in their hosts leading to a high morbidity and mortality. The consequence is a tremendous suffering on the part of men and livestock besides the significant financial burden in the agricultural and healthcare sectors. An environmentally stimulated and tightly controlled expression of virulence factor genes is of fundamental importance for streptococcal pathogenicity. Bacterial small non-coding RNAs (sRNAs) modulate the expression of genes involved in stress response, sugar metabolism, surface composition, and other properties that are related to bacterial virulence. Even though the regulatory character is shared by this class of RNAs, variation on the molecular level results in a high diversity of functional mechanisms. The knowledge about the role of sRNAs in streptococci is still limited, but in recent years, genome-wide screens for sRNAs have been conducted in an increasing number of species. Bioinformatics prediction approaches have been employed as well as expression analyses by classical array techniques or next generation sequencing. This review will give an overview of whole genome screens for sRNAs in streptococci with a focus on describing the different methods and comparing their outcome considering sRNA conservation among species, functional similarities, and relevance for streptococcal infection.

## Introduction

Bacterial small non-coding RNAs (sRNAs) play a fundamental role in the regulation of virulence factor genes in pathogenic bacteria (Papenfort and Vogel, 2010). Overall, there is still a lot more information available about riboregulators in Gram-negative model organisms, e.g., *Escherichia coli*, *Salmonella*, and *Helicobacter pylori*, compared to Gram-positive pathogens. The importance of sRNAs for pathogenesis in streptococci has been already acknowledged, but intensive functional studies are still missing. As a first step, a number of genome wide screenings for sRNAs have been performed in streptococci. Before we describe the sRNA analyses efforts in different species, we would like to introduce the significance of streptococci as causative agent of disease in human and livestock, focusing on those species, which have been subjected to sRNA screening.

According to Bergey’s Manual of Systematic Bacteriology streptococci are one of 17 genera belonging to the facultatively anaerobic Gram-positive cocci (Facklam, 2002). The genus *Streptococcus* includes a large number of individual species (>100). Typing and differentiation historically relied on haemolytic phenotypes during growth of the species on blood agar plates, designating isolates as β-hemolytic and non-β-hemolytic. This classification system was introduced by work of Schottmüller and Brown in the early 1900 [reviewed in (Facklam, 2002)]. A more refined and sophisticated classification was introduced by pioneering work of Rebecca Lancefield at Rockefeller University New York in 1933. She demonstrated specific carbohydrate “group” antigens to be discriminative in a serological typing scheme (Lancefield, 1933). The current classification and revision of the *Streptococcus* genus is based on 16S rRNA gene sequencing.

Prominent streptococcal species belonging to the ß-hemolytic group are *Streptococcus pyogenes*, *S. agalactiae, S. dysgalactiae, S. equi, S. canis and S. iniae. S. pneumonia, S. suis, S. intermedius, S. mutans,* and species of the *S. salivarius*-, *S. anginosus*-, and *S. mitis*-group are typically non-β-hemolytic (Facklam, 2002; Krzysciak et al., 2013). Humans and animals are major hosts for the above mentioned species, which occur mainly as physiological flora of skin, throat, upper respiratory tract, and intestine of their hosts. Some of them are rather opportunistic pathogens, thriving only in the background of ill or immunocompromised humans and animals. The pathogenic streptococcal species can be further classified into three distinguishable groups: common pathogenic streptococci causing infections in humans, opportunistic commensals, and zoonosis pathogens. The latter can cause disease in animals and humans or are transmitted as primarily animal pathogens to humans during farming and otherwise intensive contacts (Krzysciak et al., 2013). *S. mutans*, *S. intermedius*, *S. canis*, *S. sanguinis*, *S. salivarius,* and *S. gordonii* are opportunistic commensals causing caries, spleen, and brain abscesses, soft tissue and urinary tract infections, bacteremia, bone infections, pneumonia, meningitis, sepsis, and endocarditis, respectively. *S. suis* is currently the most prominent zoonosis pathogen among the streptococci, causing severe invasive, and mostly lethal infections in swine and humans (Fulde and Valentin-Weigand, 2013). The following part gives brief introductions into those streptococcal species for which studies and global screens for sRNAs have been performed.

### Streptococcus pneumoniae

This species can be found as commensal and colonizer of the human nasopharynx, however, under favorable conditions can cause local infections which can easily progress into life-threatening invasive diseases (most common: bacteremia and meningitis). Between 10 and 20% of adults and up to 40% of children are colonized by pneumococci, most likely building the basic pathogen reservoir for transmission and causing community acquired pneumonia. According to WHO 1.2 million infants aged below 5-years die due to pneumonia per year (data from the CDC Atlanta). Next to this infantile risk group, people aged above 65-years bear a higher risk for pneumococcal infections, which is a clear hint for a correlation of declining immune fitness and susceptibility toward peumococcal infection. A major virulence factor is the polysaccharide capsule, allowing serological distinction of over 90 capsule serotypes. Seven, 13, and 23 -valent capsule polysaccharide based conjugate vaccines are available and proved to be very efficient, however, serotype displacement in colonization phenotypes and in particular rising antibiotic resistance rates in this naturally competent species highlight pneumococci as dangerous pathogens. For the interested reader a recent and excellent review of pneumococci and their pathogenesis was published by Gamez and Hammerschmidt (2012).

### Streptococcus pyogenes

*Streptococcus pyogenes* (group A streptococci according to Lancefield scheme; GAS) is an exclusively human pathogen responsible for an extraordinary array of different diseases. *S. pyogenes* infections in immunocompetent hosts range from mild, mostly locally restricted, and self-healing diseases (pharyngitis, impetigo, pyoderma) affecting mainly skin and mucosal membranes (Cunningham, 2008; Walker et al., 2014) to severe and life-threatening invasive disease manifestations, e.g., necrotizing fasciitis and streptococcal toxic shock syndrome. The latter are associated with high morbidity and mortality rates in affected patients. The significance of *S. pyogenes* diseases is underscored by the large global burden to the national health care systems. Data compiled in 2005 by Bisno and colleagues and Carapetis and colleagues revealed 616 million cases of pharyngitis, 111 million cases of pyoderma, and at least 517.000 fatalities due to invasive diseases and sequelae (Bisno et al., 2005; Carapetis et al., 2005; Ralph and Carapetis, 2013). Sequelae manifested after non-treated primary infections comprise rheumatic heart disease and glomerulonephritis, both severely affecting underdeveloped countries and poor communities with limited access to antimicrobial chemotherapeutics. *S. pyogenes* is still fully sensitive toward penicillin. However, increasing numbers of macrolide resistant strains are of concern (Logan et al., 2012). The virulence factor repertoire, function of many virulence factors in the pathogenesis of this species, action of transcriptional regulators, two-component regulatory systems and their networking activities, as well as pinpointing molecular evolutionary events (like IndDels) leading to increased fitness and spread of certain globally disseminated strains have all been recently reviewed (Kreikemeyer et al., 2003; Hondorp and McIver, 2007; McIver, 2009; Fiedler et al., 2010; Patenge et al., 2013; Walker et al., 2014).

### Streptococcus mutans

*Streptococcus mutans* is the most prominent species in the context of caries etiology. This species is a potent biofilm former and its sugar metabolism releases acids which act on dentin to form the typical carries-associated cavities in hosts, who do not practice proper oral hygiene. Many *S. mutans* virulence factors involved in the biofilm phenotype have been characterized in detail (Krzysciak et al., 2013). This streptococcal species does also efficiently survive in human blood and is thus involved in cases of infective endocarditis, where bacteria are part of massive vegetations on heart valve regions where blood clots rich in platelets and fibrinogen cover damaged areas. The high burden on human health and the financial strain on the healthcare systems due to caries, bring these bacteria in the focus of scientific attention, which includes discovery, and functional characterization of sRNAs.

### Streptococcus suis and Other Zoonotic Streptococci

Four major species are considered zoonosis pathogens among the streptococci, including *S. canis*, a resident of the microflora of domestic carnivores, *S. equi* sp. zooepidemicus, an opportunistic pathogen in cats, rodents, minks, monkeys, and seals, *S. iniae*, an invasive fish pathogen, and *S. suis,* a major porcine pathogen occurring worldwide (Fulde and Valentin-Weigand, 2013). *S. suis* is probably the most important zoonosis pathogen, which emerged in the media spotlight after totally unexpected severe invasive disease with toxic shock like-syndrome outbreaks reported in China, 2005 (Yu et al., 2006). A serotype two strain was identified as causative agent and new emerging type which contained a previously unknown pathogenicity island. These pathogens are directly transmitted from swine to their human host during intensive contacts of farmers with their lifestock, eating of high risk dishes, like undercooked meat, blood and intestine of animals, mainly in poor low-income, and underdeveloped countries like Southeast Asia (Fulde and Valentin-Weigand, 2013). In swine, sepsis, meningitis, arthritis, and pneumonia caused by *S. suis* lead to tremendous economic losses. In humans, meningitis is the major disease that is diagnosed after *S. suis* infection.

### Streptococcus agalactiae

*Streptococcus agalactiae* (group B streptococci according to Lancefield scheme, GBS) is an important human pathogen which is found in the urogenital tract and the lower gastrointestinal tract. Up to 40% of healthy women at reproductive age are colonized with these bacteria which are apparently part of the normal flora. However, exactly this colonization site is a sincere risk for pathogen transmission to neonates during labor and birth canal passage (Dando et al., 2014). Infected neonates can develop so called early onset disease, including sepsis, pneumonia, and meningitis. This occurs in about 5000 new-borns in the US annually (Gibbs et al., 2004). Early onset diseases are associated with a 5% mortality rate. In underdeveloped countries neonate infection and killing rates are thought to be much higher, since inefficient health care systems do not provide effective mother pre-screening programs, monitoring, intensive care, and treatment options (Johri et al., 2013). Moreover, due to a majority of home births, the actual death toll numbers cannot be taken into consideration in the official statistics. Manifestation of GBS disease in elderly and immunocompromised hosts, associated with high morbidity and mortality rates, include skin and soft tissue infections, bacteremia, pneumonia, osteomyelitis, and infections of the urinary tract (Edwards et al., 2005). Unlike *S. pyogenes*, *S. agalactiae* can infect ruminants causing mastitis (Keefe, 1997). Many *S. agalactiae* virulence factors have quite some structural and functional similarities with their counterparts expressed by *S. pyogenes* and are mostly well characterized (Lindahl et al., 2005; Kreikemeyer et al., 2011). Moreover, transcriptional regulation of *S. agalactiae* virulence genes resembles mechanisms seen in other streptococcal species, a fact recently reviewed (Rajagopal, 2009; Patenge et al., 2013).

### sRNAs in Bacteria

The actual virulence of the streptococcal species described above depends on a set of specific virulence factors, which first allows the bacteria to colonize and invade the host organism and then to survive and proliferate in the hostile environment. In the course of a successful infection, bacteria have to respond to the challenging conditions at the infectious site and to the host defense mechanisms by the coordinated expression of the appropriate virulence factor genes. Bacterial adaptation to environmental changes through the regulation of gene expression has been studied intensively since the middle of the 20th century. The research focus was on the role of proteins influencing the activity of the transcriptional machinery including transcription factors, two component systems (TCSs), and sigma factors. During the first decade of this century, it became clear that RNAs serve as important regulatory molecules in eukaryotes as well as in prokaryotes. Among them, miRNAs, sRNAs, long noncoding RNAs, and riboswitches have been investigated in all three domains of life.

In bacteria, the importance of sRNAs as a distinct class of gene regulators is well established by now. First, the high number of regulatory RNAs that was found in many bacteria was unexpected (Brantl, 2009; Narberhaus and Vogel, 2009; Waters and Storz, 2009). Soon, it became evident that many diverse processes were controlled by bacterial sRNAs, including stress response, sugar metabolism, biofilm formation, and surface composition (Vanderpool and Gottesman, 2005; Gottesman et al., 2006; Heidrich et al., 2006; Gorke and Vogel, 2008; Gogol et al., 2011; Sharma et al., 2011; Mika and Hengge, 2013). Moreover, several sRNAs with housekeeping functions were identified, which are highly conserved throughout bacteria, e.g., tmRNA, 6S RNA, and RNase P (Brantl, 2009). In pathogenic bacteria, regulatory RNAs are involved in host–microbe interactions and lifestyle adaptation by controlling virulence gene expression and the general stress response (Papenfort and Vogel, 2010; Caldelari et al., 2013).

There are different classes of regulatory RNAs covering distinct modes of function. On the one hand, *cis*-acting RNAs are contained within 5′-untranslated regions (5′-UTRs) of coding transcripts. Usually, the secondary structure of the respective 5′-UTR is changed in response to an environmental stimulus, e.g., temperature in the case of RNA-thermometers or ligand binding in the case of riboswitches. As a consequence, translation initiation is inhibited or premature transcription termination occurs (Klinkert and Narberhaus, 2009; Bastet et al., 2011; Serganov and Nudler, 2013).

Another group of sRNAs is transcribed independently and functions via *cis*- or *trans*-antisense base pairing. *Cis*-acting antisense sRNAs are encoded on the opposite strand of their respective target gene. The high sequence complementarity to their target RNA leads to a very strong and specific binding. A typical example is the toxin-antitoxin system type I in bacteria, in which a *cis*-acting sRNA represses the expression of a toxic hydrophobic peptide gene by base-pairing (Brantl, 2012). In several studies, a high level of antisense transcription was detected in a variety of bacteria, with a subsequent processing by RNAse III occurring predominantly in Gram-positive bacteria (Lasa et al., 2012; Lybecker et al., 2014).

In contrast, *trans*-acting sRNAs are encoded in a location elsewhere in the genome and show a short and imperfect complementarity to their target RNAs. The consequence is a lower binding strength and target specificity that goes hand in hand with the ability to control more than one target gene. Highly complex regulatory networks are built through the interaction of sRNAs with many different targets (Papenfort and Vogel, 2009). Manifold molecular mechanisms belong to the regulatory repertoire of *trans*-acting sRNAs. Some act as repressors of translation and/or destabilize mRNA transcripts while others activate and/or stabilize target mRNAs (Frohlich and Vogel, 2009; Podkaminski and Vogel, 2010; Thomason and Storz, 2010; Storz et al., 2011).

To understand how sRNAs are able to regulate a multitude of different target mRNAs the binding regions need to be investigated. Interference with translation of target mRNAs is not restricted to the ribosome binding site (RBS) and the start codon. In *Salmonella*, translational repression by binding of GcvB to conserved C/A-rich sequences within but also upstream of the shine-dalgarno sequence of several target mRNAs was detected, indicating that repression is not solely achieved by masking the RBS but also by blocking translational enhancer sequences (Sharma et al., 2007). Furthermore, binding of the sRNA RybB to the 5′ coding region of *ompN* was also shown to repress translation in *Salmonella* (Bouvier et al., 2008). Similarly, in *Bacillus subtilis*, binding of SR1 to a region 100 nucleotides downstream from the *ahrC* RBS inhibits translation initiation by induction of structural changes downstream from the RBS (Heidrich et al., 2007). A distinct mechanism has been described for the CRISPR RNAs in *S. pyogenes*, which are involved in RNA maturation and work in concert with a specialized protein family, the Cas-proteins. In this adaptive bacterial immune system, foreign DNA is recognized and eliminated. Therefore, the presence of complementary regions in the RNA is necessary to induce specific processing of the target sequences (Deltcheva et al., 2011).

Regulation by sRNAs is not restricted to mRNA binding. There are many examples in the literature where sRNA molecules bind to and influence proteins, typically by sequestration of a factor involved in transcription or translation (Babitzke and Romeo, 2007). The function of many sRNAs in Gram-negative bacteria and in some Gram-positive species is dependent on the molecular chaperon Hfq (host factor Q-beta phage). The Sm-like protein is involved in RNA folding and facilitates sRNA–mRNA interaction (Peng et al., 2014). Not all sRNAs of a given species require Hfq for their function and in some model organisms, including streptococci, no *hfq* homologue could be detected at all (Rieder et al., 2012).

Recently, a further level of complexity in gene regulation could be demonstrated in two Gram-positive pathogens. In *Enterococcus faecalis* and *Listeria monocytogenes, eut* genes are responsible for ethanolamine utilization. A riboswitch binding to vitamin B_12_ regulates the transcription of an sRNA. In the absence of vitamin B_12_, the sRNA is synthesized and binds to the two-component system response regulator EutV. Sequestration of EutV inhibits the ethanolamine dependent activation of *eut* gene expression (DebRoy et al., 2014; Mellin et al., 2014). This two-step regulatory mechanism involving a riboswitch that controls the expression of a sRNA in combination with a two-component system, allows the integration of two environmental signals: the presence of vitamin B_12_ and of the substrate ethanolamine. Consequently, only in the presence of both molecules required for ethanolamine utilization, *eut* gene expression is initiated.

**FIGURE 1:**
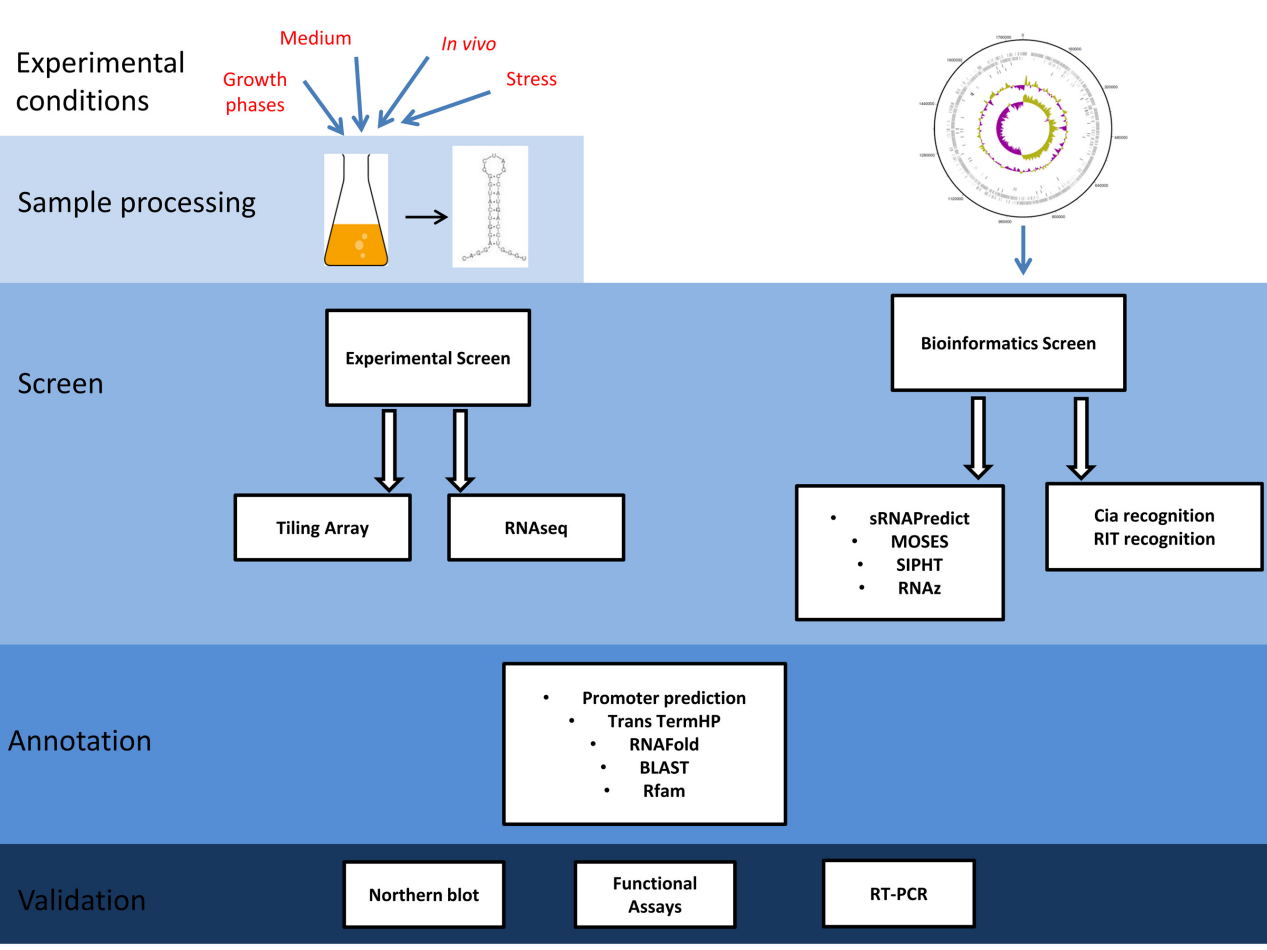
**Schematic of the work flow for the experimental and bioinformatics screens used for the detection of small non-coding RNA (sRNAs) in streptococci**. Experimental Screens: bacteria were grown under different conditions. Total RNA was prepared and processed by size exclusion and/or enrichment techniques. Samples were reverse transcribed and the resulting cDNA libraries were used for tiling arrays or next generation sequencing (RNAseq). Hybridization signals or sequence reads, respectively, were statistically analyzed for data quality and the expression level was assessed. Transcriptional start sites, sRNA length, orientation, secondary structure, sequence conservation, sRNA function, and potential targets were predicted by additional software tools (BLAST, Basic Local Alignment Search Tool; Rfam, RNA families database). Finally, likely sRNA candidates were validated by Northern blot or reverse transcription PCR (RT-PCR) and a subset of sRNAs was further characterized by deletion analyses, functional assays, and *in vivo* infection models. Bioinformatics screens: Fully sequenced reference genomes of the organism of interest were either analyzed using sRNA prediction algorithms (MOSES, modular sequence suite; SIPHT, sRNA identification protocol using high-throughput technology; RNAz, Fast and reliable prediction of non-coding RNAs) or alternatively, known recognition sites for sRNA related proteins were used as a signature to detect novel sRNA candidate genes. Prediction of sRNAs was followed by annotation and validation analogous to the experimental screens.

To fully understand bacterial pathogenesis, virulence gene regulation by sRNAs has to be taken into account. In recent years, many screens have been conducted for the identification of sRNAs in Gram-positive bacteria. For a subset of these sRNAs, the respective targets could be verified experimentally (Mraheil et al., 2010; Brantl and Bruckner, 2014). The knowledge about the regulatory function of sRNAs in streptococci has been summarized in three recent review articles (Le and Charpentier, 2012; Patenge et al., 2013; Miller et al., 2014). Here, an overview will be given of different screening methods that have been applied for the analyses of sRNA expression detection and genome-wide bioinformatics prediction in streptococcal species (**Figure 1**). If available, examples for the role of sRNAs in virulence related gene expression control will be described.

## Streptococcus pneumoniae

### Screening for sRNAs Controlled by the CiaR/H Regulatory System in *S. pneumoniae*

A typical feature of sRNAs is the cross-communication with protein-mediated gene expression regulation pathways. In many cases sRNA gene expression is controlled by transcriptional regulators or, *vice versa*, sRNAs influence the expression of regulator genes. Thus, it is not surprising that the first sRNA genes identified in *S. pneumoniae* were part of the CiaRH two-component regulatory circuit, which is involved in competence and virulence. In a transcriptional mapping study, a direct repeat motif, TTTAAG-N5-TTTAAG, was detected in three promoters that were known to be directly regulated by the response regulator CiaR. CiaR binding to the repeat region was shown in gel-shift assays and the importance of the repeat for transcriptional activation by CiaR was demonstrated by promoter mutation experiments. Subsequently, the *S. pneumoniae* genome was analyzed by motif and pattern searches and 15 promoters were identified that were controlled by CiaR. Of those, the five strongest promoters were found to drive the expression of sRNAs, designated csRNAs (*cia*-dependent sRNAs; Halfmann et al., 2007; **Table 1**). All five csRNAs showed a high degree of sequence conservation and structural similarity, marked by two stem-loops separated by about 40 unpaired nucleotides. The presence of the respective csRNAs was verified by Northern blot analyses. Deletion analyses revealed that csRNA4 and csRNA5 are involved in autolysis control, whereas the other three csRNAs did not affect autolysis behavior. Competence was not influenced by any of the csRNAs (Halfmann et al., 2007). Within the first loop of the csRNAs1-3, and csRNA5, a CCUCCU motif is conserved, which could serve as an anti-SD sequence, hinting toward inhibition of translational initiation by blocking of ribosome binding. By sequence comparison, csRNAs were detected in other *S. pneumoniae* strains and in closely related streptococci like *S. mitis* and *S. sanguinis* (Halfmann et al., 2007).

**Table 1:** Global small non-coding RNA (sRNA) screens in streptococci – techniques and outcome.

Species	Strains	Screening method	Number of sRNAs	Unknown sRNAs	Validated (method)	Length (nt)	Reference
*S. pneumoniae*	TIGR4	Whole genome tiling array	50	36	13 RT-qPCR	74–480	Kumar et al. (2010)
*S. pneumoniae*	TIGR4	sRNAPredict2	63				Livny et al. (2006)
*S. pneumoniae*	D39	sRNAPredict/Northern blot	10	9	10 Northern blot	64–400	Tsui et al. (2010)
*S. pneumoniae*	TIGR4	494 sequencing, size fractionated RNA	88	77	17 RT-PCR/Northern blot	52–391	Acebo et al. (2012)
*S. pneumoniae*	TIGR4	Illumina sequencing, size fractionated RNA	89	56	41 Northern blot 4 RT-PCR	31–400	Mann et al. (2012)
*S. pyogenes*	MGAS5005	sRNAPredict2	42			60–452	Livny et al. (2006)
*S. pyogenes*	MGAS2221 (M1T1 GAS)	Whole genome, intergenic tiling array	53	40	20 Northern blot	50–800	Perez et al. (2009)
*S. pyogenes*	GAS M49 591	Whole genome, intergenic tiling array	55	42	6 Northern blot and RT-PCR	49–364	Patenge et al. (2012)
*S. pyogenes*	MGAS315	sRNAPredict, RNAz, eQRNA/Northern blot	45	7	14 Northern blot and RT-PCR	94–282	Tesorero et al. (2013)
*S. pyogenes*	SF370 M1 GAS	Differential RNAseq					Deltcheva et al. (2011)
*S. sanguinis*		SIPHT	34				Livny et al. (2008)
*S. mutans*		SIPHT	18				Livny et al. (2008)
*S. mutans*		Illumina sequencing, size fractionated RNA	900	msRNAs	7 RT-qPCR	15–26	Lee and Hong (2012)
*S. mutans*	UA159, TW1 (Δ*ccpA)*	RNAz prediction/Illumina sequencing	114				Zeng et al. (2013)
*S. suis*	P1/7	Differential RNAseq	29		5 deletion analyses		Wu et al. (2014)
*S. agalactiae*	NEM316	*In silico* model based on RIT signatures	197		26 RT-PCR/10 Northern blot		Pichon et al. (2012)

In a follow-up approach, csRNAs were searched systematically in streptococci: if the ciaRH system was conserved throughout streptococci, *cia*-dependent sRNAs might be present as well. A BLAST search (Altschul et al., 1990) using the 5 csRNA sequences originally identified in *S. pneumoniae*, revealed all 5 csRNAs in all *S. pneumoniae* strains tested and some hits with limited similarity in other streptococcal species (Marx et al., 2010). To be able to detect potential csRNAs with a low sequence similarity to the *S. pneumoniae* csRNA sequences, the intergenic regions (IGRs) of 14 streptococcal genomes were screened for CiaR controlled promoters followed by terminators, using motif search, and TransTermHD (Kingsford et al., 2007; Marx et al., 2010). Fifty eight candidate csRNA genes were predicted, representing 40 different csRNA types. The overall sequence similarity between the various csRNAs was low. Only short sequence stretches of the csRNAs were conserved, including the region consisting of the anti-SD sequence plus anti-startcodon and a conserved nonamer, [A/C]UCCUAAA[A/C], located at the 5′ region of the csRNA or following the first stem–loop, respectively. Since the molecular chaperone Hfq is missing in streptococci (Valentin-Hansen et al., 2004), one possible function of this second conserved stretch is a protein binding region for the interaction with an alternative protein. The number of csRNAs in the various species varied from two to six, but each csRNA predicted in one strain could be detected in all other strains by BLAST. The expression of the predicted csRNAs was verified by Northern blot analyses in *S. sanguinis*, *S. mitis*, and *S. oralis* (Marx et al., 2010). In this screen, a high number of Cia-dependent promoters was detected allowing the refinement of the CiaR-binding sequence using WebLogo (Crooks et al., 2004). Based on this information the consensus sequence for CiaR binding was changed to NTTAAG-N5-TTTAAG (Marx et al., 2010). Taken together, csRNAs are present in all streptococci but not in unrelated Gram-positive bacteria. While the sequence conservation between csRNA types is limited, all csRNAs seem to belong to the CiaR regulon. The elucidation of the regulatory mechanism of the individual csRNAs will shed light on their role within the CiaR/H regulatory circuit.

### Global Screenings for sRNAs in *S. pneumoniae*

In a bioinformatics sRNA screening using sRNAPredict2, 63 sRNA candidate genes were detected in *S. pneumoniae* TIGR4 (Livny et al., 2006). The results of this study served as basis of a systematic validation approach in serotype 2 D39 (Tsui et al., 2010). Therefore, a BLAST search was conducted, in which 40 candidate genes were detected with more than 90% identity to the initially predicted sequences. Northern blot validation confirmed the expression of csRNA1 and of nine novel sRNAs in serotype 2 D39 (**Table 1**). Five of the sRNA genes showed differential expression dependent on the growth phase or upon stimulation with competence stimulatory peptide (Tsui et al., 2010).

Using whole genome tiling microarrays, 50 sRNAs were identified in *S. pneumoniae* serotype 4 TIGR4 (Kumar et al., 2010). The overlap with the 63 sRNAs candidates from the data set of the sRNAPredict2 study (Livny et al., 2006) was very low. Only eight sRNAs were detected with both approaches, four of which had been verified before by Northern blot (Tsui et al., 2010). The five csRNAs identified in *S. pneumoniae* R6 (Halfmann et al., 2007) could be detected in *S. pneumoniae* TIGR4. The expression of 13 candidate sRNA genes was validated by quantitative reverse-transcriptase PCR (qRT-PCR). All candidate sRNA genes were highly conserved within pneumococcal strains. Twenty five sRNAs were conserved in closely related streptococci (*S. mitis, S. gordonii, S. sanguinis*) but not in other streptococci (*S. pyogenes, S. mutans, S. bovis*) and only six sRNA sequences were conserved in other Gram-positive species. Functional predictions for 14 sRNAs were possible using the Rfam database (Griffiths-Jones et al., 2003; Nawrocki et al., 2015), whereas for 36 sRNA gene candidates no function could be assigned, which made them likely candidates for novel sRNAs. Eight sequence motifs present in the pneumococcal sRNAs were identified using MEME SUITE (Bailey et al., 2009). Two of the motifs were detected in the five homologs of the csRNAs identified in *S. pneumoniae* R6 (Halfmann et al., 2007), underlining the conservation of csRNAs in pneumococci. For two motifs no putative function could be predicted by Rfam and the other four motifs were specific for different types of *cis*-regulatory sRNAs (Kumar et al., 2010).

Another screening for sRNA candidates in *S. pneumoniae* serotype 4 TIGR4 was performed employing a 454 pyrosequencing approach (Acebo et al., 2012). Total RNA was size-fractionated and 5S rRNA-depleted prior to cDNA library preparation to enrich the sRNA population in the sample. 135 contig sequences were overlapping the 5′-end (57) or the 3′-end (78) of an ORF and a subset of those might represent *cis*-regulatory RNAs. As candidates for putative sRNAs encoded within an IGR, 88 sequences were identified. Eighteen candidates corresponded with sRNAs detected in the previous TIGR4 tiling array study, six of which had been validated by RT-PCR (Kumar et al., 2010). Of the 63 sRNAs assigned by sRNAPredict2 (Livny et al., 2006), eight candidates were detected in this study (Acebo et al., 2012). Three of those overlapping sRNAs were also present in the tiling array data set (Kumar et al., 2010). From the previously identified five CiaR-dependent sRNAs, csRNA3 and csRNA5 were detected, whereas csRNA1, csRNA2, and csRNA4 appeared to be absent. Due to the high sequence homology of the csRNAs to each other, the authors assumed that sequence reads were incorrectly assigned to csRNA3 during mapping, masking the presence of csRNA1, csRNA2, and csRNA4. Functional prediction using the Rfam database (Griffiths-Jones et al., 2003) led to the assignment of three candidates as housekeeping RNAs (RNase P, tmRNA, and 6S RNA) and eight candidates as *cis*-regulators, including riboswitches and ribosomal protein gene leader sequences. Seventy seven sRNAs did not show any homology to any known RNA family and were considered novel sRNAs in *S. pneumoniae* (Acebo et al., 2012). Target prediction using TargetRNA (Tjaden et al., 2006) followed by functional analyses led to the conclusion that *srn206* is involved in CSP-dependent competence regulation in *S. pneumoniae*, probably by interaction with the ComD pathway. From 44 sRNAs ≥100 nt in lenght, seven were specific for *S. pneumoniae* and 37 were conserved in other streptococci, indicating a species- and genus-specific conservation of pneumococcal sRNAs (Acebo et al., 2012).

TIGR4 and three isogenic TCS mutants were analyzed in a whole-genome RNAseq experiment with the aim to identify sRNAs involved in pneumococcal virulence (Mann et al., 2012). The mutant strains carried mutations in the response regulator genes of the TCSs GRR, Cbpr, and VncR, respectively. Total RNA of each strain was size-fractionated (<200 nt), individually sequenced, and the resulting data were pooled. For the prediction of sRNA genes, a prokaryotic promoter prediction program (University of Groningen) and TransTermHP (Kingsford et al., 2007) were employed. The analysis revealed 89 putative sRNAs. Of those, 56 were novel and 33 had been identified before in the studies described above. Expression of 41 sRNA candidate genes was verified by Northern blot, four sRNAs were confirmed by RT-PCR, and 10 sRNAs had been confirmed in previous studies. Comparison of the different strains revealed 24 sRNA candidates that were not detectable in the parental TIGR4 strain but were expressed in at least one of the TCS mutant strains. In accordance with the other sRNA screens in pneumococcus, more than 90% of the identified sRNAs were conserved in *S. pneumoniae*, 11 were conserved amongst streptococci, and 17 amongst other Gram-positive bacteria. A sequence motif search utilizing MEME SUITE (Bailey et al., 2009), revealed five different motifs that were conserved in several sRNA candidates. Sequence analyses using the Rfam database (Griffiths-Jones et al., 2003) predicted six putative sRNA sequences to be *cis*-acting riboswitches. In this study, the influence of sRNAs on pneumococcal virulence was studied using a murine model of infection. From the sRNAs that were confirmed by Northern blot, 15 candidates were picked for deletion analyses and eight of the sRNA deletion mutants were attenuated in the progression of sepsis. Furthermore, pathogenesis was investigated by Tn-Seq fitness determination (Opijnen et al., 2009). As a result, a total of 72 sRNAs were predicted to influence bacterial fitness in specific host niches: 28 in the lung, 26 in the nasopharynx and 18 in the blood (Mann et al., 2012).

## Streptococcus pyogenes

Until recently, in *S. pyogenes* (GAS) a low number of sRNAs had been described and reported to be involved in the control of pathogenesis (PEL, FasX, RivX, and CRISPR; Kreikemeyer et al., 2001; Mangold et al., 2004; Roberts and Scott, 2007; Deltcheva et al., 2011). A bioinformatics screening using sRNAPredict2, identified 42 putative sRNA genes in GAS (Livny et al., 2006). However, the three sRNAs already known at the time (PEL, FasX, RivX) were not included. Since then, several whole genome expression screenings have been undertaken to allow a more comprehensive view of the sRNA landscape in GAS.

In MGAS2221, representing the highly virulent M1T1 GAS clone, a custom whole genome intergenic tiling array approach was used to detect sRNAs expressed in the exponential phase of growth in complex medium (Perez et al., 2009). From 40 putative sRNAs identified in this study, only seven had been detected in the previous sRNAPredict2 study (Livny et al., 2006). Additionally to the sRNA genes, 13 small RNA candidates were identified with *cis*-regulatory or other typical RNA functions, including seven riboswitches and two CRISPR elements. Sixteen sRNAs and four examples of the other small RNAs (CRISPR-1, the riboswitches metK2 and serS, and 4.5S RNA, the RNA component of the signal recognition particle) were verified by Northern blot analyses. Sequence conservation over GAS genomes was tested with all 75 sRNA genes detected in the tiling array and the former bioinformatics study. Twelve sequenced GAS genomes were used for the analysis and a majority of 62 sRNAs was present in all of the genomes tested (Perez et al., 2009).

**FIGURE 2:**
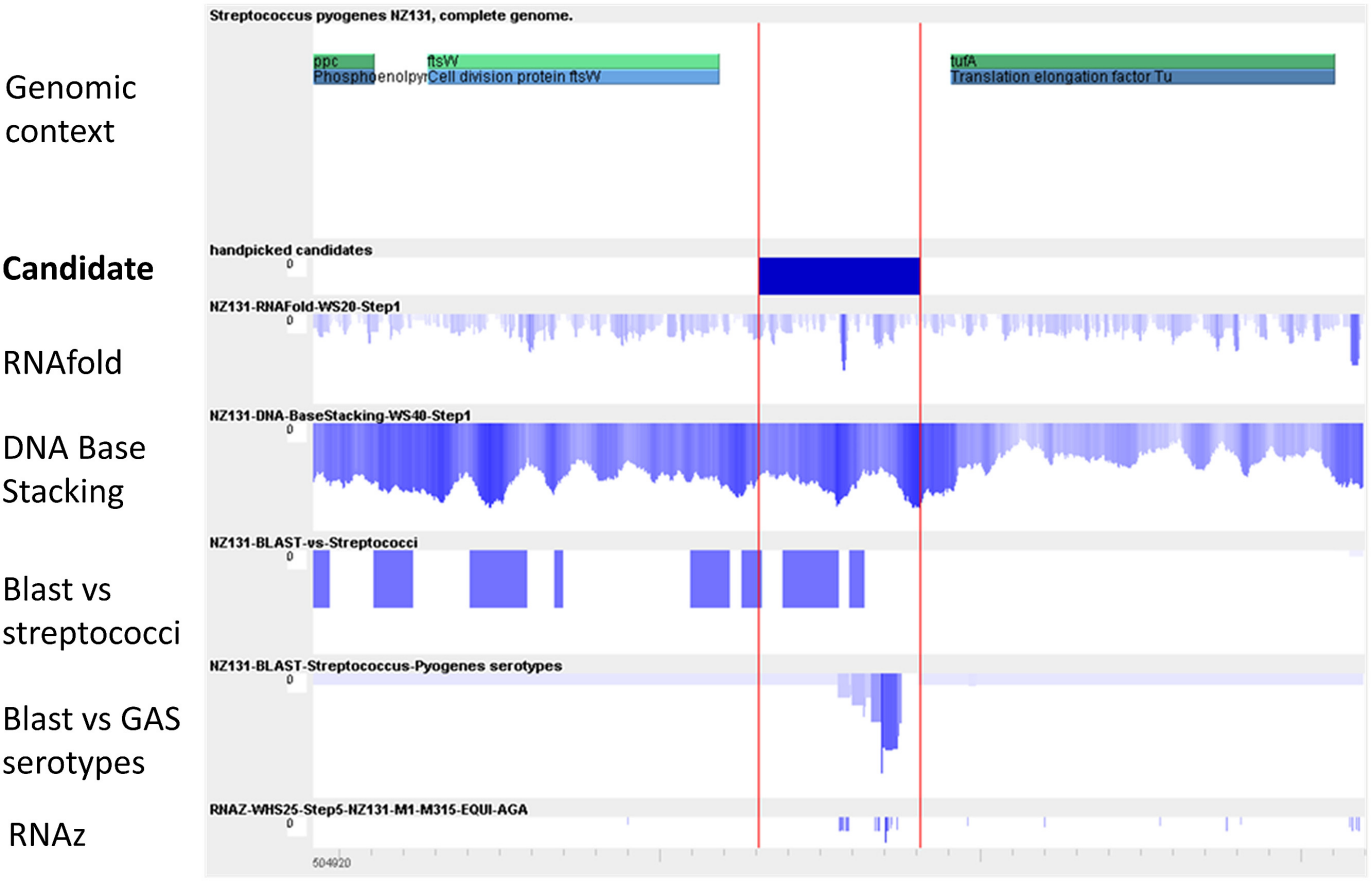
**Visualization of the key modules used by MOSES for intergenic sRNA detection**. Modified from Raasch et al. (2010). The first row shows the annotation as provided by NCBI. In the second row, the handpicked candidate is pictured for comparison with the prediction data from the various algorithms. In the third row, an RNA-fold minimal energy profile is depicted. A peak indicating a strong secondary structure is visible within the intergenic region (IGR). The next two rows demonstrate conservation over streptococci and over *S. pyogenes* genomes, respectively, as determined by BLAST. In the last row, predictions using RNAz are shown.

In a similar study our laboratory identifed sRNA genes in GAS M49 (Patenge et al., 2012). Whole genome intergenic tiling arrays were used to detect transcriptional activity throughout growth in chemical defined media (CDM). In this screen, 55 putative sRNA genes were detected. Of those, 42 sRNAs were novel, but for 14 of the novel sRNA candidates a function could be predicted using the Rfam database (Griffiths-Jones et al., 2003), including several *cis*-regulatory RNAs, one tmRNA, an endoribonuclease (bacterial RNase P), and the RNA subunit 4.5S RNA of the SRP. Conservation of the sRNA genes was high. From 55 sRNAs, 53 were present in all GAS serotypes tested by BLAST analyses. For six sRNA candidates, the transcriptional start site was determined by 5′ RACE, the expression was validated by RT-PCR and Northern blot analysis, and expression patterns were compared between growth phases and with growth in complex medium. The data from the tiling arrays were compared to the results of two bioinformatics prediction programs, MOSES (Raasch et al., 2010), and sRNAScanner (Sridhar et al., 2010). The modular sequence suite (MOSES) was developed, because of the high demand to combine various sRNA prediction modes in one convenient software tool, to achieve a higher reliability of the predicted data (**Figure 2**). In accordance with the sRNA screens in streptococci described above, the overlap between the expression results and the computer predictions was very low.

Computational screening, followed by validation of expression employing Northern blot and RT-PCR, was performed in the M3 serotype strain MGAS315 (Tesorero et al., 2013). To optimize the accuracy of the bioinformatics approach, three computational algorithms were combined: sRNAPredict (Livny et al., 2006), eQRNA (Rivas and Eddy, 2001), and RNAz (Washietl et al., 2005). All candidates that were predicted by any two of the algorithms were considered sRNA candidates. Sequences located directly upstream of ORFs were considered *cis*-regulatory elements and excluded from further analysis. In the exponential growth phase, 14 sRNAs were detected by Northern blot analysis: FasX, the *sagA* transcript, and 12 novel sRNAs. Further investigation by RNAseq and sequence analyses revealed that three of those were not *trans*-acting sRNA. Two were *cis*-regulatory elements and one appeared to be the bacterial ribonuclease P. Another two sRNAs had been detected in previous screens (Perez et al., 2009; Patenge et al., 2012), seven sRNA candidates represented novel putative *trans*-acting sRNAs.

Differential RNA sequencing (dRNAseq) has been recently introduced in a study of *H. pylori* (Sharma et al., 2010). The method allows the detection of transcriptional start sites and has been proved a powerful tool for the detection of small transcripts, including sRNAs. In GAS SF370, a M1 serotype, dRNAseq has been used to screen specifically for the expression of CRISPR elements in GAS. This approach led to the identification of a novel class of RNAs, *trans*-activating CRISPR RNA (tracrRNA), which includes sequences complementary to the repeat regions of crRNAs and is involved in their maturation (Deltcheva et al., 2011).

## Oral Streptococci

Posttranscriptional gene regulation in oral bacterial species has been recently reviewed (Merritt et al., 2014). With a bioinformatics tool developed for the kingdom-wide prediction and functional annotation of bacterial sRNA genes, SIPHT [sRNA identification protocol using high-throughput technologies, (Livny et al., 2008)] 18 sRNA genes were predicted in *S. mutans* and 34 sRNA genes in *S. sanguinis*. To date, no functional information is available for those putative sRNA genes. As described in the chapter about *S. pneumoniae*, the strong conservation of CiaR and CiaR activated promoters among streptococci was exploited to identify csRNAs in many species including oral streptococci (Halfmann et al., 2007): 3 in *S. mutans*, 6 in *S. gordonii*, 6 in *S. sanguinis*, 5 in *S. oralis,* and 5 in *S. mitis* strain B6 and 2 in strain SF100. Expression of the csRNAs was further verified by Northern blot analysis in *S. mitis, S. oralis*, and *S. sanguinis*. The functional mechanism of the csRNAs in oral streptococci needs further investigation.

In *S. mutans* the presence of microRNA-size small RNAs (msRNAs) has been analyzed by a deep sequencing approach (Lee and Hong, 2012). More than 900 putative msRNAs could be detected with a size of 15–26 nt. From the candidates with the highest abundance, seven were validated by qRT-PCR. Even though the function of those msRNAs is still cryptic, they might play a role in msRNA gene expression regulation or may be secreted into the surrounding saliva and take part in inter-species regulatory processes by influencing host gene expression (Lee and Hong, 2012).

Members of the oral microbiome are highly dependent on the availability of carbohydrates. Uptake systems and carbohydrate catabolic pathways are strictly regulated by the carbon catabolite repression, which is controlled in *S. mutans* by the catabolite control protein A (CcpA; Abranches et al., 2008). Differential expression of coding genes and of RNAz predicted sRNAs in response to carbohydrate availability was investigated by an RNAseq approach in *S. mutans* UA159 (WT) and TW1 (*ccpA* mutant) strains (Zeng et al., 2013). By comparing the two strains and growth conditions in the presence of glucose versus galactose, 10 sRNAs were found to be differentially expressed under these conditions. The function and relevance of these sRNA candidates needs to be determined.

In *S. mutans* UA159 another bioinformatics approach using four different programs, sRNAPredict (Livny et al., 2005), sRNASVM (Saha and Raghava, 2006), SIPHT (Livny et al., 2008), and “Oral Pathogens Non-Coding Small RNA Prediction” (www.oralgen.lanl.gov/_index.html), led to the detection of 334 sequences, 40 of which were predicted by at least two of the programs. In this study, only the L10-Leader was characterized further and found to be highly abundant in *S. mutans* UA159 by Northern blot analyses.

## Streptococcus suis

A differential RNA-sequencing approach has been used to identify sRNAs from *S. suis* (Wu et al., 2014). To understand adaptive transcriptional regulation in *S. suis* strain P1/7 was grown in rich medium, pig blood, or cerebrospinal fluid, and RNAseq was performed. Twenty nine sRNAs were identified. Conservation of 10 sRNAs was shown in other *Strepotcoccus* species. Five sRNAs were functionally characterized. Deletion of the sRNA candidates did attenuate virulence of the mutants in a zebrafish infection model. Deletion of three sRNAs led to a higher sensitivity toward killing by pig blood. The respective sRNA genes were influencing the expression of several virulence factor genes, including genes involved in capsule synthesis (Wu et al., 2014).

## Streptococcus agalactiae

As described above, a variety of different *in silico* sRNA prediction tools is available, based on a combination of features including sequence homologies, secondary structure predictions, calculation of stability, prediction of transcriptional start sites, and detection of rho-independent termination sites. The methods employed so far were not suited to predict antisense-RNAs (asRNAs) from genomic data. However, transcriptome data from several genera implicated that antisense transcriptional activity is common in bacteria and is likely to play a role in virulence control (Toledo-Arana et al., 2009; Lorenz et al., 2010; Sharma et al., 2010). In a study from Pichon et al. (2012) an algorithm was developed that allows the prediction of sRNAs as well as asRNAs. The method is based on the detection of RIT-associated signatures. RIT is a recognition site for sRNA-binding proteins involved in the termination process of sRNA genes in *E. coli*, which is also found in Gram-positive bacteria (Pichon and Felden, 2005). From 197 predicted sRNAs in *S. agalactiae* NEM316, 26 were validated by RT-PCR and 10 of those showed a strong signal in Northern blot analyses. Genomic comparison showed that none of the sRNAs detected in *S. agalactiae* with this screening method were present in *S. pyogenes*, implying high species specificity. In overexpression experiments, three of the candidate RNAs could be shown to regulate the gene expression of adjacent target genes (Pichon et al., 2012).

## Conclusion

Over the last decade, a growing number of sRNA screens both computational and experimental has been performed in streptococci. Taken together, an approximate number of at least 100 sRNAs should be expected per genome (**Table 1**). It is likely that more sophisticated techniques will uncover even more relevant small RNA molecules, because it is estimated that there are several 100 sRNAs per bacterial genome. The size of the determined sRNAs lies between 40 and 500 nt, which is in accordance with the results from Gram-negative genera (Storz et al., 2011). Sequence comparisons revealed that streptococcal sRNAs are highly conserved between closely related organisms on the genus or species level and that some are conserved over streptococci. In contrast to Gram-negative examples, sRNAs are not conserved over rather unrelated Gram-positive genera. Exceptions are the small house keeping RNAs, including tmRNA and 4.5S RNA, with defined functions different from gene expression regulation.

In all species discussed here, the overlap between expression data and bioinformatics prediction data sets was low. Beside the issue of false positive predictions from both approaches, there are several reasons for this phenomenon. While an efficient prediction algorithm could principally detect every sRNA gene within a given genome due to its sequence and structural features, experimental screens are always dependent on the expression of a given sRNA. Expression – and therefore sRNA detection – depends on the strain, growth conditions, growth phase, availability of certain metabolites, or the presence of stress, or other stimulatory signals. For example, in *S. pyogenes*, the overlap between the results of two different tiling array screens were low, due to expression differences between the M serotypes used and the growth conditions analyzed in the respective studies (Perez et al., 2009; Patenge et al., 2012). Additionally, the sRNA gene expression is required to reach at least the detection level of the technique employed. Many of the screens discussed here were using genomic tiling arrays or were combining bioinformatics prediction with RT-PCR or Northern blot analyses. With all approaches discussed, no initiation or termination sites can be determined. Thus, the screens were usually accompanied by further computational analyses, e.g., promoter prediction programs and rho-independent terminator prediction.

An answer to the limitations of these methods is the application of high throughput screening methods like next generation sequencing, which become more and more available and affordable. With deep sequencing approaches, a high number of conditions can be studied in parallel by simply pooling the respective samples. A demonstrative example is the exploration of the *Salmonella* transcriptional landscape (Kroger et al., 2012). CRISPR expression in *S. pyogenes* (Deltcheva et al., 2011) and the adaptive responses of the transcriptome in *S. suis* (Wu et al., 2014) have been investigated using dRNAseq. This technique allows the mapping of transcriptional start sites and distinguishes native RNA species from their mature forms (Sharma et al., 2010). It is used for the annotation of ORFs and operons and the identification of novel transcripts, including sRNAs. Therefore, the application of dRNAseq is a promising tool for the comprehensive determination of independently transcribed sRNAs in streptococci. However, the more complex the data sets become, the more challenging the data evaluation will be. An overview over the analyses of bacterial RNAseq data has been given by McClure et al. (2013).

The development of advanced bioinformatics screening methods tries to overcome the obstacles of sheer sequence comparison. The aim is to find more candidates but also to combine more specific characteristics of sRNAs for more stringent results, e.g., screening for conserved RNA secondary structures rather than conserved sequences. In the Java-based framework MOSES, several algorithms are included in one tool to consider sequence conservation and secondary structure among other specifics (Raasch et al., 2010). Tesorero et al. (2013) were screening for sRNAs in *S. pyogenes* by combining several different computational applications, sRNAPredict, RNAz, and eQRNA followed by validation of candidates by Northern blot. In the future the combinatorial approach will be supported by machine learning, allowing the algorithms to learn from data by building models throughout the screening. An overview over sRNA prediction methods and future developments is given by Li et al. (2012).

To better understand the regulatory influence of sRNAs in bacteria and specifically to understand the impact of sRNAs on the virulence in streptococci, data from transcriptional regulation and posttranscriptional regulation must be integrated. The use of publicly available expression compendia in combination with sequenced-based predictions could be used to build sRNA-target interaction models and to analyze the impact of sRNAs on the transcriptional network (Ishchukov et al., 2014). Furthermore, the host–microbe interaction could be studied by using dual-seq approaches involving streptococci and their respective hosts as has been proposed by Westermann et al. (2012).

## Conflict of Interest Statement

The authors declare that the research was conducted in the absence of any commercial or financial relationships that could be construed as a potential conflict of interest.
